# A Method for Monitoring the Working States of Drainage Tubes Based on the Principle of Capacitance Sensing

**DOI:** 10.3390/s20072087

**Published:** 2020-04-08

**Authors:** Kai Luo, Wenpu Shi, Yinghao Chen, Bo Wang, Jialin Yao, Xing Yang

**Affiliations:** 1 The State Key Laboratory of Precision Measurement Technology and Instruments, Department of Precision Instrument, Tsinghua University, Beijing 100084, China; m17810264557@163.com (K.L.); yaojialin92@gmail.com (J.Y.); 2 School of Mechatronics and Automobile Engineering, Yantai University, Yan Tai 264005, China; swp636793@163.com (W.S.); chenyinghao0203@gmail.com (Y.C.); wangbo-1314@mail.tsinghua.edu.cn (B.W.); 3 School of Materials Science and Engineering, University of Science and Technology Beijing, Beijing 100083, China

**Keywords:** drainage tube, drainage state, blockage, interdigital capacitance

## Abstract

The real-time monitoring of the working status of drainage tubes is crucial for successful surgical drainage and for informing clinicians of the drainage conditions of patients at different stages, to enable objective diagnosis and treatment. In this study, a method for monitoring the drainage condition of drainage tubes was proposed. The method was based on the principle of capacitance and was developed by analyzing the major states of drainage tubes in the process of drainage. Meanwhile, the principle of interdigital capacitance monitoring drainage was analyzed, and an interdigital capacitance device for the real-time monitoring of the working status of drainage tubes was designed. Ultimately, an experimental system for drainage simulation was established on the basis of the interdigital capacitance device and method for drainage monitoring. Results showed that the interdigital capacitance device for drainage monitoring can identify unobstructed or blocked drainage tubes effectively in real time. The device has a hydrophobic surface, so its electrodes do not undergo electrolysis and pollution due to adhesion. Hence the proposed capacitance-based method for monitoring the working states of drainage tubes has good application prospects in the postoperative drainage of abdominal and thoracic cavities.

## 1. Introduction

Surgical drainage is an important routine operation in surgery, which is widely applied in pre- and postsurgical treatment. It is performed to drain effusions, such as hematocele, bile, and pancreatic juices, out of the body or into other body parts to prevent infection, diminish inflammation, accelerate the concrescence of wounds, and reduce complications [1,2,3,4]. Drainage devices are necessary for surgical drainage and the most common drainage devices are drainage tubes, given their simple structures and low cost. Drainage tubes have the advantages of safety and minimal invasion, which effectively ensure drainage [5,6,7,8,9,10,11,12,13].

There are several problems in using drainage tubes. First, drainage tubes are easily blocked by pus, blood clots, stones, tissue fragments, and floccules. Second, the small intestine or lung tissue has the possibility of blocking the drainage tube orifice during continuous negative pressure suction using the drainage tubes. Moreover, the bending of drainage tubes may result in blockages and failure of drainage. Blockages of the drainage tube may act as a trigger for hydrops and infection in the drainage site, which even threaten the patient’s life if proper remedial measures are not taken [14,15,16].

Timely inspection is the common clinical method used to solve the above problems, where the states of drainage tubes should be monitored and observed regularly. However, it is hard to observe blockages and requires patients and medical staff to examine drainage tubes regularly. Checking the working state of drainage tubes for unconscious or weakly conscious patients is more difficult and will increase the workload of medical staff. Therefore, the intelligentization of drainage tubes should be improved to realize the real-time monitoring of the statuses of drainage tubes with less human intervention and examination, and to enable medical staff and patients to find and resolve drainage tube blockages in time. Studies have focused on the improvement of the intelligentized drainage tubes by means of sensors. For example, Chen et al. studied and applied a thoracic drainage system with a pressure sensor for the real-time detection of changes in pleural pressure to help clinical doctors judge the different periods of recovery of the chest [17]. Linsler et al. reported that an intelligent drainage tube can be used to detect intracranial pressure during cerebrospinal fluid drainage [18]. However, research on real-time supervision devices for monitoring the flow condition of drainage tubes has not been found. Therefore, the various working states of drainage tubes were investigated in this study. An interdigital capacitance device for drainage monitoring based on the principle of capacitance sensing was proposed and analyzed. The device was fabricated by printed circuit technology and chemical vapor deposition technology.

## 2. Principle and Method

**Figure 1:**
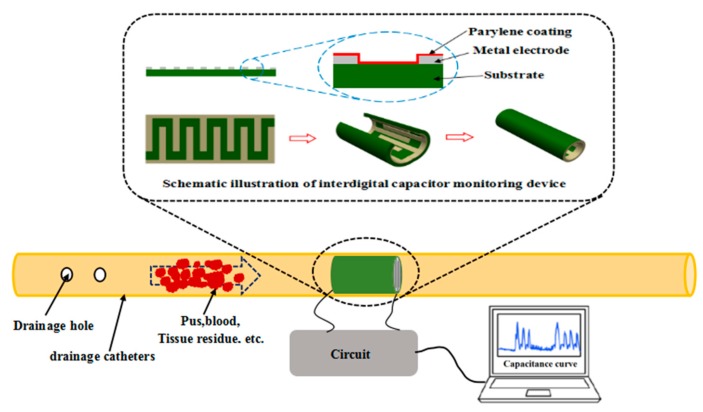
Schematic and detection principle of the interdigital capacitance device for drainage state monitoring.

The sensing unit is the most important component for realizing intelligent drainage monitoring, and the appropriate sensing methods must be selected in accordance with the characteristics of the drainage to acquire the information of the various working states of the fluids in drainage tubes. Relative permittivity changes or capacitive changes have been used for monitoring the environments [19]. The capacitive sensing method is more suitable for monitoring drainage state by comprehensively considering various factors, such as usage environment, stability, and sensitivity, and the sensing principle is as follows. The capacitance of the parallel plate capacitor is described as follows:

(1) $$C=\varepsilon_{0}\varepsilon_{r}\frac{A}{d}$$

where *ε*_0_ and *ε*_r_ are the vacuum dielectric constant and relative dielectric constant, respectively; *A* is the effective area of the parallel plate electrode; and *d* is the distance between two electrode plates. When a parallel plate capacitor is installed in the drainage tube, the effective area of parallel plate electrode *A* and the distance between the two electrode plates *d* are unchanged, meanwhile, *ε*_0_ is a constant. As the drainage fluid flows through the capacitor, *ε*_r_ will be changed under different flow conditions of drainage, thus, *C* will change accordingly. Therefore, the information of drainage status can be inferred from monitoring the *C* of the sensor element. The above is the basic principle underlying the capacitance-based monitoring of drainage conditions. Among sensor technologies, the interdigital sensors have a wide range of applications, such as humidity and gas sensors [20], and biosensors [21,22,23]. To simplify the structure and provide a convenient installation, an interdigital capacitance device for drainage monitoring was designed, as shown in Figure 1, which has several advantages such as low cost, simple structure and process, and strong antijamming ability.

If the interdigital capacitance device was placed in the drainage tube, and the interdigital electrodes must be in direct contact with the drainage fluid during drainage. In this condition, the interdigital electrode will generate electrolyzation which will reduce the working life of the monitoring device. In addition, gas generated from electrolyzation can pose hidden dangers to patients. Furthermore, the adherence of tissue residues and blood clots during drainage to the interdigital electrode result in some errors of measurement results. Therefore, the electrode surface was covered with an insulating layer of Parylene C (poly(chloro-p-xylylene)) to prevent the electrode from electrolyzing. Meanwhile, the adherence of tissue residue and blood clots to the electrode was reduced because Parylene C is hydrophobic [24,25,26,27]. Thus, the electrode can resist pollution and is cleaned easily when tissue residues, blood clots, and other drainage materials flow through the surface of the interdigital capacitance device.

**Figure 2:**
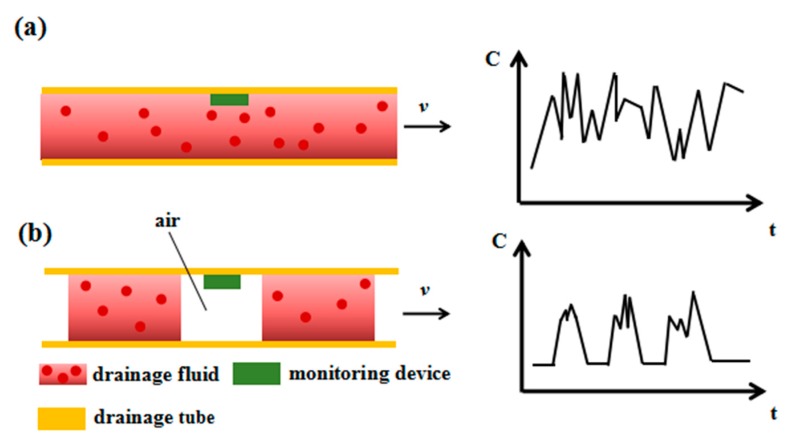
Schematic diagram of the flow state and the output of the sensor when the drainage tube is unblocked. (**a**) The working state of the drainage tube and the corresponding capacitance change curve when the entire drainage pipeline is unblocked and the liquid is continuously discharged. (**b**) The working state diagram of the drainage tube and the corresponding capacitance change curve of the sensor in the conditions of the entire pipeline is unobstructed and the amount of drainage liquid is small, where the liquid and air are intermittently discharged.

When using the drainage tubes, the drained fluids may under some circumstances be returned to the human body. We can add a check valve or use negative pressure to realize drainage in order to avoid backflow. In this article, we only analyze and discuss the normal situation of drainage fluid being discharged out of the human body. The drainage fluid in the tube is complex and includes various kinds of substances in different states, such as liquids, droplets, liquid columns, and gas–liquid–solid mixtures. The working states of the drainage tube can be classified into two major types: unobstructed state and obstructed state. The unobstructed state is mainly divided into two situations: the first situation of drainage with high fluid volume and the second situation of drainage with low fluid volume. Given the large amount of liquid in the first situation, the drainage fluid is discharged constantly and fills the tube at a certain flow rate. Drainage fluid is present at low amounts and is discharged intermittently with gas in the second situation. The flow state and monitoring principle of the drainage fluid in the unobstructed tube are shown in Figure 2. Figure 2a illustrates the situation wherein drainage fluid discharge is large and unobstructed. In this state, the drainage fluid is continuously discharged and fills the drainage tube at a certain flow rate. At this point, the tube is filled with liquid, and a small amount of gas and solid are mixed in the liquid, so the dielectric constant does not change much, and the measured *C* value fluctuates within a small range. Figure 2b presents the situation wherein drainage fluid discharge is small and drainage is unobstructed. In this state, air and drainage fluid are discharged intermittently where the dielectric constant of air is unchanged and the dielectric constant of the drainage fluid is variational. Therefore, the *C* value measured by the drainage monitoring device exhibits a situation in which a steady state intersects with a pulse state.

**Figure 3:**
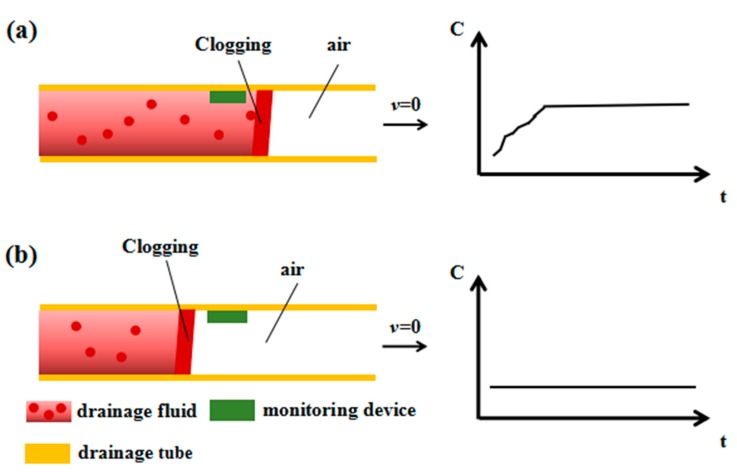
Schematic diagram of the flow state and the output of the sensor when the drainage tube is blocked. (**a**) The working state diagram of the drainage tube and the corresponding capacitance change curve of the sensor when the pipeline is blocked and the blocking position is behind the sensor. (**b**) The working state diagram of the drainage tube and the corresponding capacitance change curve of the sensor when the pipeline is blocked and the blocking position is in front of the sensor.

Drainage fluid does not flow when the drainage tube is obstructed. Different blocking positions result in different *C* values. There are two sites of blockage in the drainage tube, the rear and the front of the monitoring position. In this condition, the flow state and monitoring principle in the drainage tube are shown in Figure 3. Figure 3a illustrates the situation wherein the blockage is in the rear of the monitoring device. The drainage fluid near the monitoring location will accumulate until it does not flow at all, and *ε*_r_ will change to a certain value and remain stable. Thus, the *C* value measured by the drainage monitoring device will also change to a certain value and remain unchanged. Figure 3b shows the situation wherein the blockage is in the front of the monitoring device. In this case, the drainage fluid does not flow, and the drainage monitoring device is always in air. Thus, *ε*_r_ does not change, and the *C* value measured by the drainage monitoring device will not change.

## 3. Design and Fabrication of the Interdigital Capacitance Device for Drainage Monitoring

**Figure 4:**
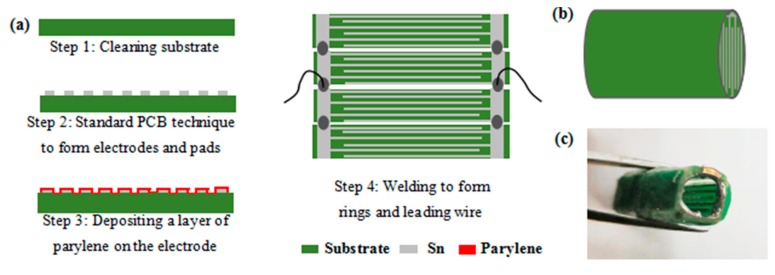
(**a**) Flowchart of the manufacturing process of the capacitive device for drainage monitoring. (**b**) Schematic of the approximate cylinder-shape capacitive device for drainage monitoring. (**c**) Photo of the capacitance device for drainage monitoring.

The fabrication of the interdigital capacitance drainage monitoring device is simple and mainly by virtue of printed circuit technology and chemical vapor deposition technology. More details are shown in Figure 4a. Steps 1 and 2: solder joints and interdigital electrodes were constructed on the substrate by using standard PCB (Printed Circuit Board) technology. Step 3: the insulating Parylene C layer was deposited on the surface of the interdigital electrode with a thickness of 1 µm through chemical vapor deposition. Step 4: the interdigital capacitive drainage monitoring device was made by bending the PCB with electrodes into an approximate cylinder shape and soldering the leads. These processes are commonly used in manufacturing. Therefore, the monitoring device has low manufacturing costs and can be produced easily in large quantities. In this study, the electrodes of the interdigital capacitance monitoring device were constructed with approximate cylindrical shapes and installed on the internal wall of the drainage tube to facilitate the comprehensive detection of drainage fluid flow through the annular wall of the drainage tube. The assembled approximate cylinder-shape interdigital capacitance monitoring device is shown in Figure 4b, and the photo of the interdigital capacitive device for drainage monitoring is shown in Figure 4c.

## 4. Experiment and Result Analysis

**Figure 5:**
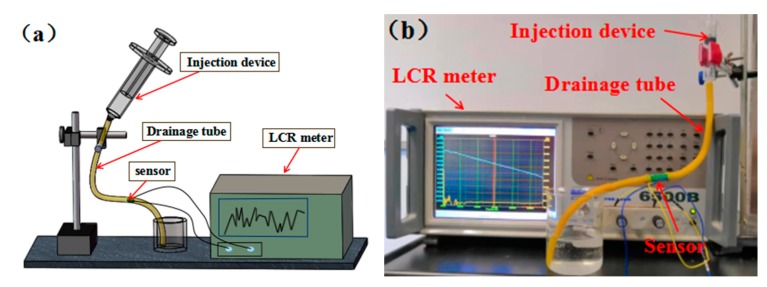
(**a**) Schematic diagram of the simulation experiment system. (**b**) Photo of the simulation experiment system.

Several drainage working states corresponding to Figure 2 and Figure 3 have been experimentally simulated in this study. Experimental results were analyzed in accordance with the above monitoring principles. Normal saline solution was used to simulate drainage fluid, and a syringe was used to simulate drainage sites in the human body. An impedance analyzer (Waynekerr WK6500B) was used to obtain the capacitance information of the interdigital capacitance sensing device. The schematic of the simulation experiment system is shown in Figure 5.

The experiments could be divided into four cases which correspond to the four states shown in Figure 2 and Figure 3.

**Figure 6:**
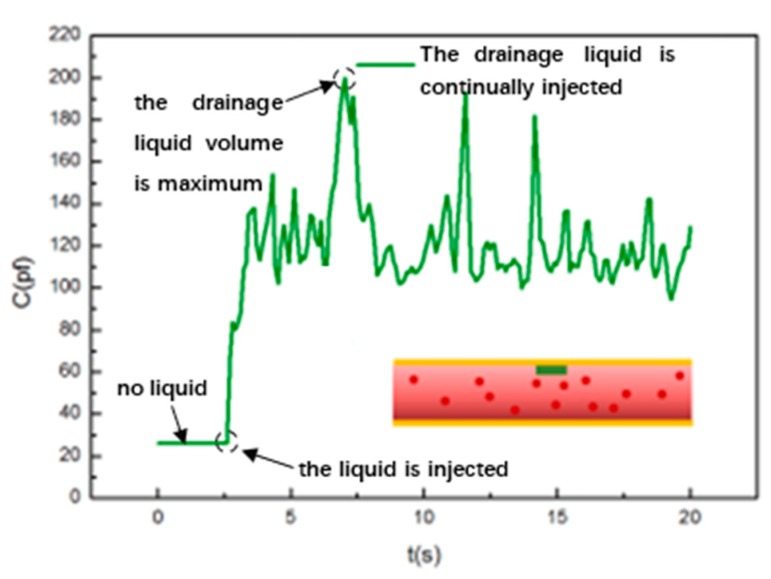
The capacitance output curve of the sensor when the entire pipeline is unobstructed and the drainage liquid is continuously discharged.

Case (1) (corresponds to Figure 2a): the liquid is continually injected in the pipeline to simulate the working state of the drainage tube when a large amount of body fluid is continuously discharged of the patient drainage. In this case, the entire pipeline is unobstructed. Figure 6 shows the experimental results. In this situation, the “drainage fluid” flows in the pipeline with some fluctuations. The ratio of the “drainage fluid” and the air in the pipeline is always changing, that is, the dielectric medium of the drainage monitoring capacitive sensor fluctuates all the time, so the dielectric constant εr of the sensor also fluctuates all the time. As a result, the capacitance value C of the sensor are kept at a large value with some fluctuations.

**Figure 7:**
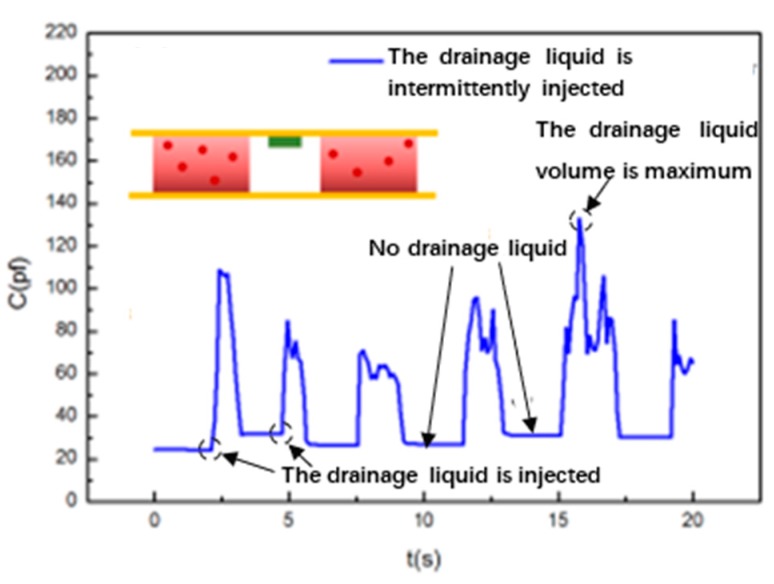
Capacitance change curve diagram of the sensor monitoring the drainage tube’s working state when the entire pipeline is unobstructed and liquid and air are intermittently discharged.

Case (2) (corresponds to Figure 2b): we intermittently inject liquid into the pipeline to simulate the reduction state of the drainage fluid. In this case, the drainage fluid is sporadic in the drainage tube and the drainage liquid and air are alternately discharged. The experimental results are shown in Figure 7. In this case, the large difference between the dielectric constant of the drainage fluid and the dielectric constant of air results in the pulsating output of the drainage monitoring sensor.

**Figure 8:**
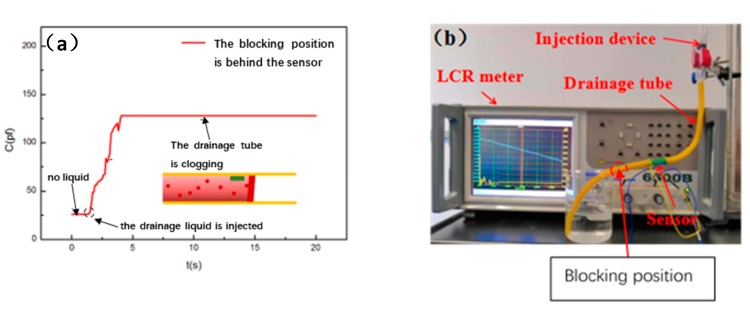
(**a**) Capacitance change curve of the sensor where the drainage tube is blocked and the blocking position is behind the sensor. (**b**) Photo of blockage position in simulation experiment.

Case (3) (corresponding to Figure 3a): In this case, we plug a plug in the drainage tube’s downstream position, which is behind the sensor, to simulate the working state of the drainage tube’s clogging position being behind the sensor. In this condition, the drainage fluid will accumulate at the position behind the sensor and will fill the part of the drainage tube behind the sensor. So, εr will gradually increases and will stabilize after reaching a certain value. Therefore, the output curve of the drainage monitoring sensor will also increase to a certain value and remain stable, as shown in Figure 8.

**Figure 9:**
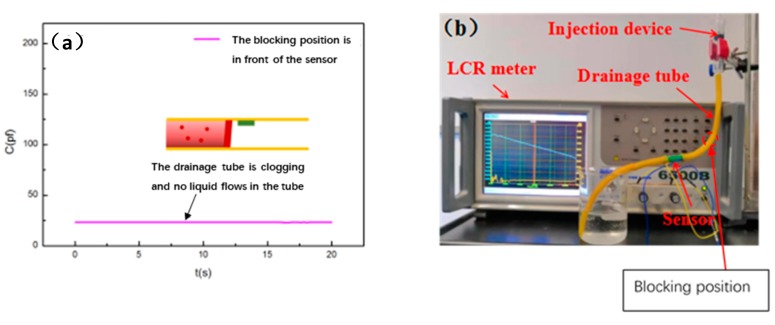
(**a**) The sensor’s output curve corresponding to the working state where the drainage tube is blocked and the blocking position is in front of the sensor. (**b**) Photo of blockage position in simulation experiment.

Case (4) (corresponding to Figure 3b): In this case, we plug a plug in the drainage tube’s upstream position, which is in front of the sensor, and simulate that the blocking position is located in front of the sensor. In this condition, the drainage fluid will not flow through the drainage monitoring sensor and will be always in the air. Thus, the output of the drainage monitoring sensor will almost be a constant where air is the dielectric layer, and the curve is shown in Figure 9.

The above experiment demonstrated that when the drainage tube was unobstructed, the C value measured by the capacitance drainage monitoring sensing device constantly changed with time, and the C value fluctuated slightly with a large base value when a large amount of body fluid is continuously discharged. When drainage fluid flow was intermittent, the output of the drainage monitoring sensor would be pulsatile with a small base valve. When the drainage tube was obstructed at different positions, the drainage tube was filled with different media, and the *C* values measured by the drainage monitoring sensor differed. In either case, however, the *C* value eventually remains almost steady. Therefore, in actual practice, a threshold of the C value can be set in accordance with the situations of drainage and the experience of professional doctors. When the drainage tube is blocked, an alarm will be triggered if the *C* value of the capacitance sensor for drainage monitoring reaches the threshold. The above experiments show that the proposed interdigital capacitance sensing device can effectively monitor the status of drainage tubes during drainage in practical applications.

## 5. Conclusions

This study proposed a method and designed an interdigital capacitance sensing device for monitoring the state of drainage tubes. This method was based on the principle of capacitance sensing, and the interdigital capacitance device was designed to monitor the states of drainage tubes and was fabricated with a simple method. We conducted a simulation experiment of drainage using the interdigital capacitance sensing device. Experimental results revealed that the continuous discharge and intermittent discharge of the drainage fluid will cause the *ε*_r_ to fluctuate or change. The change in *ε*_r_ reflected the corresponding changes in *C* values measured by the capacitance sensing device for drainage monitoring. This result proved that the capacitive monitoring method can effectively obtain the state information of the drainage tube on the basis of the changes in *C* value caused by the changes in *ε*_r_. The capacitance-based method for drainage monitoring can identify whether a drainage tube is unobstructed or obstructed during drainage, which should have significance in application for doctors to comprehensively analyze the drainage situation of patients at different stages and make an objective diagnosis and treatment.
